# Increased dietary intake of ultraprocessed foods and mitochondrial metabolism alterations in pediatric obesity

**DOI:** 10.1038/s41598-023-39566-9

**Published:** 2023-08-03

**Authors:** Serena Coppola, Lorella Paparo, Giovanna Trinchese, Andrea Margarita Rivieri, Antonio Masino, Anna Fiorenza De Giovanni Di Santa Severina, Mariapina Cerulo, Maria Escolino, Assunta Turco, Ciro Esposito, Maria Pina Mollica, Roberto Berni Canani

**Affiliations:** 1 Department of Translational Medical Science, University Federico II, Naples, Italy; 2 ImmunoNutritionLab at CEINGE Advanced Biotechnologies, University Federico II, Naples, Italy; 3 Department of Biology, University Federico II, Naples, Italy; 4 European Laboratory for the Investigation of Food-Induced Diseases, University Federico II, Naples, Italy; 5 Task Force for Microbiome Studies, University Federico II, Naples, Italy

**Keywords:** Obesity, Paediatric research

## Abstract

The increased intake of ultraprocessed foods (UPFs) in the pediatric age paralleled with the risen prevalence of childhood obesity. The Ultraprocessed Foods in Obesity (UFO) Project aimed at investigating the potential mechanisms for the effects of UPFs in facilitating pediatric obesity, focusing on the direct role of advanced glycation end-products (AGEs) on mitochondrial function, the key regulator of obesity pathophysiology. We comparatively investigated the daily dietary intake of UPFs, energy, nutrients, dietary AGEs [Nε -(carboxymethyl)lysine (CML), Nε -(1-carboxyethyl)lysine (CEL), and Nδ -(5-hydro-5- methyl-4-imidazolon-2-yl)-ornithine (MG-H1)] in 53 obese patients and in 100 healthy controls visiting the Tertiary Center for Pediatric Nutrition of the Department of Translational Medical Science at the University of Naples “Federico II”. AGEs skin accumulation and mitochondrial function in peripheral blood mononuclear cells (PBMCs) were also assessed. A higher intake of UPFs and AGEs, energy, protein, fat, and saturated fatty acids was observed in obese patients. Obese children presented significantly higher skin AGEs accumulation and alterations in mitochondrial metabolism. PBMCs from healthy controls exposed to AGEs showed the same mitochondrial alterations observed in patients. These findings support the UPFs role in pediatric obesity, and the need for dietary strategies limiting UPFs exposure for obesity prevention and treatment.

## Introduction

Pediatric obesity is one of the most common worldwide public health challenges, requiring great efforts of the clinical research into its prevention and management^1^. In the last decades, increased consumption of ultraprocessed foods (UPFs) paralleled with the risen prevalence of childhood obesity^2^. UPFs are industrial formulations of processed food that generally contain additives and other substances to make the final product palatable and more appealing, including advanced glycation end-products (AGEs), dominant compounds generated during late stages of the Maillard reactions occurring between carbonyl and amino groups^3^. Several published data have provided evidence regarding the detrimental effects of UPFs and their compounds AGEs on human health, and linked their dietary intake with the occurrence of non-communicable diseases including obesity^4^. Evidence have shown that AGEs can impair mitochondrial function resulting in abnormal reactive oxygen species (ROS) production and ATP level reduction^5–7^. Mitochondria play a crucial role in energy metabolism, whose dysfunctions can lead to obesity and metabolic alterations^8^.

The Ultraprocessed Food in Obesity (UFO) Project was launched to investigate the potential mechanisms of the UPFs effects in facilitating pediatric obesity, investigating the direct role of major components of UPFs (i.e., the AGEs).

We comparatively investigated the daily dietary intake of UPFs, energy, nutrients, major dietary AGEs [Nε -(carboxymethyl)lysine (CML), Nε -(1-carboxyethyl)lysine (CEL), and Nδ -(5-hydro-5- methyl-4-imidazolon-2-yl)-ornithine (MG-H1)] in obese pediatric patients and in healthy controls. AGEs skin accumulation and mitochondrial function in peripheral blood mononuclear cells (PBMCs) were also assessed.

## Results

### Higher dietary UPFs intake in obese patients

53 consecutive obese pediatric patients (52.8% male, mean age 10.7 years ± 3.5 SD) and 100 healthy controls (63% male, mean age 10.3 years ± 3.5 SD) were enrolled in the study.

The participants’ features are given in Table 1.

**Table 1:** Study subjects’ features.

Variables	Healthy controls (n = 100)	Obese patients (n = 53)
Male, n (%)	63 (63)	28 (52.83)
Age, mean years (± SD)	10.3 (± 3.5)	10.7 (± 3.5)
Born at term, n (%)	83 (83)	48 (90.56)
Spontaneous delivery, n (%)	31 (31)	19 (35.84)
Birth weight, mean kg (± SD)	3.10 (± 0.58)	3.27 (± 0.49)
Breastfed for ≥ 1 month, n (%) - Duration of breastfeeding, mean m (± DS)	75 (75) 7.33 (± 8.52)	39 (73.6) 8.26 (± 8.33)
Weaning age, mean months (± SD)	5.32 (± 0.86)	5.55 (± 0.99)
Smoking during pregnancy, n (%)	14 (14)	2 (3.77)
Smoking during breastfeeding, n (%)	10 (10)	2 (3.77)
Urban living, n (%)	95 (95)	49 (92.45)
Parents/legal guardian educational level		
- Middle school, n (%)	11 (11)	7 (13.21)
- High school, n (%)	54 (54)	30 (56.6)
- University, n (%)	35 (35)	16 (30.19)
Body weight, mean kg (± SD)	37.71 (± 13.12)*	67.72 (± 26.09)
Height, mean cm (± SD)	141.3 (± 19.16)	146.1 (± 17.41)
BMI, mean kg/m^2^ (± SD)	18.25 (± 2.29)*	30.7 (± 6.79)

Continuous variables are reported as mean (± SD). Discrete variables are reported as the number and proportion of subjects with the characteristic of interest.

*SD* standard deviation, *BMI* body mass index.

*p < 0.001*.*

Subjects enrolled in the two study groups were comparable for all the demographic and anamnestic features, with the exception, as expected, for body weight and Body Mass Index values, that were significantly higher in obese patients if compared to healthy controls (67.72 kg ± 26.09 *vs.* 37.71 kg ± 13.12, *p* < 0.0001; 30.7 kg/m^2^ ± 6.79 *vs.* 18.25 kg/m^2^ ± 2.29, *p* < 0.0001).

**Figure 1:**
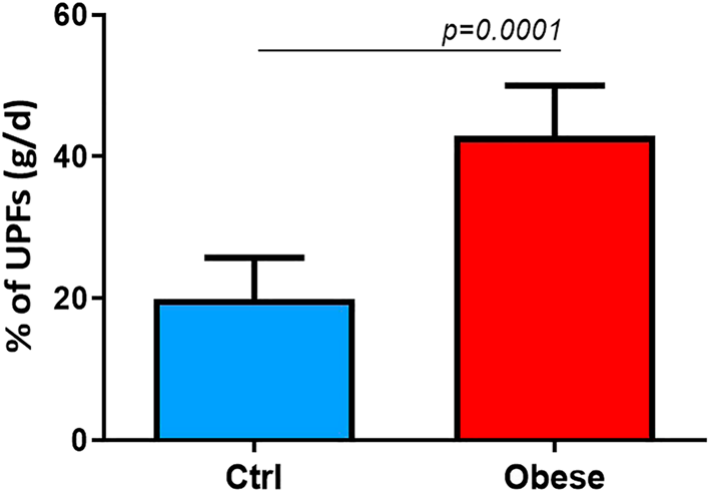
Higher dietary UPFs intake in obese patients in %. The unpaired t-test found that patients with obesity reported a significantly higher dietary intake of UPFs than healthy controls in the proportion (%) of UPFs in the total weight of food and beverages consumed (g/day). Data are expressed as median 95% CI.

**Figure 2:**
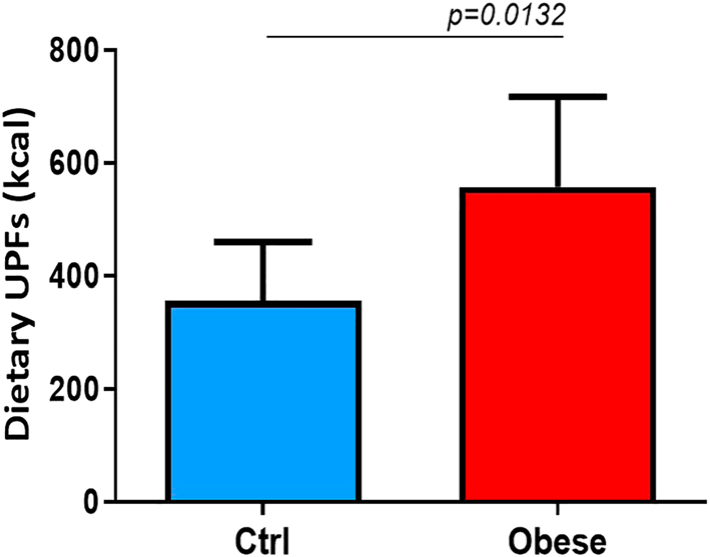
Higher dietary UPFs intake in obese patients in Kcal. The unpaired t-test found that patients with obesity reported a significantly higher dietary intake of UPFs than healthy controls and in the daily energy (Kcal) deriving from UPFs. Data are expressed as median 95% CI.

A significantly higher dietary intake of UPFs was observed in pediatric patients with obesity than controls, both in the proportion (%) of UPFs in the total weight of food and beverages consumed (g/day) [42.9% (95%CI 27–50) *vs.* 19.85% (95%CI 13.4–25.7), *p* = 0.0001], and in the daily energy (Kcal) deriving from UPFs [557.7 kcal (95% CI 433.4–717.8) *vs.* 356.5 kcal (95%CI 301.6–460.2), *p* = 0.0132] (Figs. 1 and 2).

Obese patients reported a significantly higher daily dietary intake than healthy controls of energy (1741 kcal 95%CI 1506–1871, *vs.* 1470 kcal 95%CI 1362–1608, *p* = 0.01), protein (67.71 g 95%CI 64.88–73.45 *vs.* 60.92 g 95%CI 58.34–63.82, *p* = 0.02), fat (68.26 g 95%CI 57.14–79.78 *vs.* 49.49 g 95%CI 43.72–56.07, *p* = 0.01), and saturated fatty acids (16.38 g 95%CI 11.71–21.37 *vs.*10.80 g 95%CI 8.62–13.49, *p* = 0.0009).

We observed a trend toward higher daily dietary intake of CML, CEL, and MG-H1 in obese children than healthy controls [2.7 mg (95%CI 2.1–3.4) *vs.* 2.3 mg (95%CI 2.0–2.8), *p* = 0.26; 1.9 mg (95%CI 1.6–2.3) *vs.* 1.5 mg (95%CI 1.3–1.7), *p* = 0.99; 14.5 mg (95%CI 11.8–18.7) *vs.* 10.4 mg (95%CI 9.7–11.9), *p* = 0.09].

**Figure 3:**
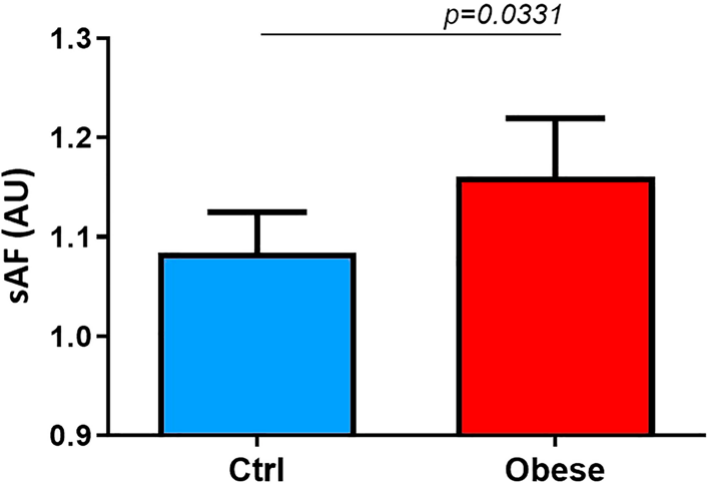
Increased AGEs skin accumulation in obese children. The unpaired t-test found that patients with obesity presented significantly higher skin AGEs accumulation compared to healthy controls. Data are expressed as mean 95% CI. **Abbreviations:** sAF, skin autofluorescence.

In addition, obese patients presented significantly higher skin AGEs accumulation compared to healthy controls (1.2 ± 0.21 *vs.* 1.1 ± 0.21, *p* = 0.03) (Fig. 3).

### Effects of AGEs exposure on mitochondrial metabolism in PBMCs

**Figure 4:**
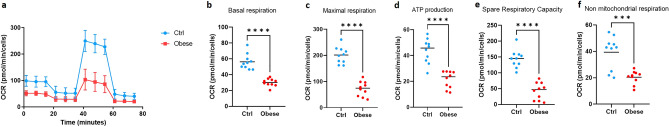
Mitochondrial respiration-linked parameters in PBMCs. Representative graph of Cell Mito Stress assay performed by Seahorse XFp analyzer is reported (**a**). Basal respiration (**b**), maximal respiration (**c**), ATP production (**d**), spare respiratory capacity (**e**), and non mitochondrial respiration (**f**) are reported. Each point in the OCR time courses is the average of three technical replicates (n = 10/group). The values are expressed as mean ± SD. The unpaired t-test has been performed to compare the groups. ***p < 0.001; ****p < 0.0001.

We investigated the mitochondrial metabolism in PBMCs collected from obese children, and from healthy controls at baseline and after AGEs exposure. Representative graph of Cell Mito Stress assay performed by Seahorse XFp analyzer is reported in Fig. 4a. At the baseline, we observed a significant reduction in basal (− 46% *vs.* healthy controls) and maximal (− 64% *vs.* healthy controls) oxygen consumption rates, and in ATP synthesis (− 50% *vs.* healthy controls), in PBMCs from obese patients compared to healthy controls, suggesting an alteration of mitochondrial metabolism, leading to impaired oxidative capacity of substrates (Fig. 4b–d). The reduction of spare respiratory capacity (SRC), a functional parameter evaluating cell competence to respond to increased energy demand or stress, was also reduced in obese children (− 70% *vs.* healthy controls) (Fig. 4e). After the addition of rotenone and antimycin A, to inhibit complexes I and III, the non-mitochondrial respiration residual resulted lower (− 48% *vs.* healthy controls) in obese children (Fig. 4f).

**Figure 5:**
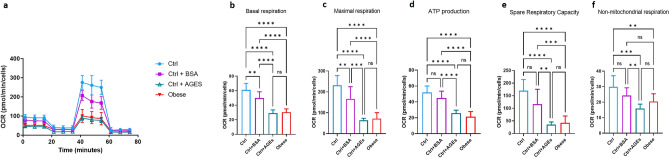
Mitochondrial respiration-linked parameters in PBMCs from healthy controls exposed to AGEs. Representative graph of Cell Mito Stress assay performed by Seahorse XFp analyzer is reported (**a**). Basal respiration (**b**), maximal respiration (**c**), ATP production (**d**), spare respiratory capacity (**e**), and non mitochondrial respiration (**f**) are reported. Each point in the OCR time courses is the average of three technical replicates (n = 7/group). The ANOVA and the Newman-Keuls post hoc test to correct for multiple comparisons have been performed to compare the groups. The values are expressed as mean ± SD. **p < 0.05;***p < 0.001; ****p < 0.0001.

Then, analyzing mitochondrial metabolism in PBMCs from healthy controls incubated with AGEs, we observed mitochondrial alterations similar to those observed in obese patients, characterized by a significant decrease of basal and maximal respiration, ATP production, SRC, and non mitochondrial respiration (Fig. 5a–f).

Finally, we measured the ratio between glutathione (GSH) and reduced GSH/oxidized glutathione (GSSG), as plasmatic biomarkers of oxidative stress. We showed that the GSH/GSSG ratio in plasma of obese patients was significantly lower compared to healthy controls [mean ± standard error (0.21 ± 0.01 *vs.* 0.32 ± 0.01, *p* < 0.0001)], indicating higher oxidative stress level.

## Discussion

In recent decades, changes in the global food system, particularly the substantial expansion of highly processed foods in the world’s food market, have been hypothesized as a contributor to the worldwide rise in childhood obesity^9^.

We confirmed a significantly higher UPFs consumption in obese pediatric patients. This data is in line with results deriving from cross-sectional^10–12^ and prospective studies conducted in pediatric populations^13–16^. These studies, mainly conducted in American countries, revealed that higher consumption of UPFs was related with obesity prevalence and increased fat mass^10–12^, and reported that the food processing has a role in body fat accumulation beyond its calorie content^13^. Furthermore, it has been showed that higher dietary intake of UPFs is associated with more rapid progression of excess weight into adolescence and early adulthood^14–16^.

Several mechanisms have been postulated for UPFs in facilitating the occurrence of obesity. Palatability and high energy density of UPFs may facilitate excessive energy intake^17^. Furthermore, high refined sugar and fat content may alter satiety signaling and may produce changes in neurocircuitry reward, leading to overconsumption and addictive-like eating behaviors^18–20^. In addition, UPFs can alter insulin response and promote the storage of excess nutrients in adipose tissue^21^.

UPFs provide high levels of the detrimental compounds AGEs, Maillard products formed during non-enzymatic reactions^22^. Animal protein, fat and saturated fatty acids foods, mostly cooked at high and dry heat, such as broiling, grilling, frying, and roasting, represent the main dietary sources of AGEs^23^. We observed a significantly higher dietary intake of energy, protein, fat, and saturated fatty acids in obese patients than controls. AGEs could be involved in the pathophysiology of obesity by altering directly protein structure and function, and by leading to an increased production of ROS and inflammatory cytokines, mainly by binding the AGEs receptor (RAGE), and ends up creating a vicious circle, exacerbating the typical chronic inflammation state of obesity^24,25^.

The AGEs–RAGE axis plays an important role in obesity, contributing to adipogenesis of preadipocytes^26^, and to adipocyte hypertrophy^27^. Preclinical studies have shown that high AGEs-diet induces obesity and related metabolic alterations compared to low AGEs-diet^28,29^. In turn, nutritional interventions aimed at reducing the dietary AGEs intake have been proved to be effective in reducing the risk of obesity and its complications in humans^30^.

We observed a trend toward higher daily dietary intake of AGEs in obese children than healthy controls. Similar results have been reported by others^31^. Furthermore, the higher consumption of dietary AGEs was associated with an increased risk to develop metabolic syndrome^32^. Unfortunately, the reference database used in these studies to quantify the dietary AGEs intake reported the content of only one AGEs (CML) in foods, determined by semi-quantitative enzyme-linked immunosorbent assay based on a monoclonal anti-CML antibody^23^.

To our knowledge, this is the first study evaluating the dietary AGEs intake in pediatric subjects with and without obesity using a reference database reporting the content of the most common AGEs (CML, CEL and MG-H1) quantified in the protein fractions of 190 foods using a highly sensitive, specific and rapid ultra-performance liquid chromatography tandem mass spectrometry method^33^.

Dietary AGEs may induce adverse health outcomes through their accumulation in human tissue^34^. We also observed a higher skin AGEs accumulation in obese pediatric patients. This result is in line with previous observations reported by others^35^.

Mitochondrial dysfunction could be involved in the pathogenesis of obesity^36^.

Another potential mechanism elicited by UPFs and their compounds AGEs in facilitating obesity, could be ascribed to their capacity to induce mitochondrial metabolism alterations^37^. AGEs could be able to increase the production of mitochondrial ROS and to decrease the ATP production^5,38,39^.

We observed alterations of mitochondrial metabolism in obese children, characterized by reduced basal and maximal respiration, ATP production, SRC, and non mitochondrial respiration. Similar mitochondrial metabolism alterations were observed in PBMCs from healthy controls incubated with AGEs, suggesting an additional mechanism elicited by dietary AGEs in facilitating pediatric obesity.

Potential limitations of our study could be related to the cross-sectional design, which do not address causality, and the relatively small sample size. Further studies, with prospective design evaluating younger and larger cohorts, could be necessary to confirm our results.

However, the present study has several strengths. Our results are consistent with previous evidence. The evaluation of the seven-day weighed food diaries using a high specific reference database provided an accurate evaluation of dietary UPFs and AGEs intake. The higher dietary AGEs exposure in children affected by obesity paralleled with a higher AGEs skin accumulation. In vitro experiments provided mechanistic data on the potential role of AGEs in facilitating the occurrence of pediatric obesity, through a negative impact on mitochondrial metabolism.

## Conclusions

Our findings support the role of UPFs exposure in the pediatric obesity pandemic. The results of the UFO study provided data on potential mechanisms for the UPFs effects in facilitating pediatric obesity (i.e., mitochondrial alterations) involving the major components of UPFs (i.e., the AGEs). These results further support the potential importance of limiting dietary UPFs exposure as additional strategy for an integrated approach for pediatric obesity prevention and treatment.

## Methods

### Study design

The UFO (Ultraprocessed Foods in Obesity) Project was an observational, case-control, single-center, pilot study designed to investigate the potential mechanisms elicited by UPFs in facilitating pediatric obesity.

### Ethics declarations

The study was approved by the Ethics Committee of the “University of Naples Federico II—A.O.R.N. Cardarelli” (Protocol n°00019173) and was performed in accordance with the Helsinki Declaration (Seoul revision, October 2008) and with the relevant European and Italian privacy regulations. Written informed consent to participate into the study was obtained by the subjects and their parents/legal guardian. The UFO Project was registered on https://clinicaltrials.gov/ with the identifier NCT05554016.

### Participants

From September 2022 to December 2022, consecutive caucasian subjects of both sexes, aged ≥ 6 and ≤ 18 years, with a diagnosis of obesity (based on a Body Mass Index (BMI) > 97th percentile for age and sex) visiting for the first time the Tertiary Center for Pediatric Nutrition of the Department of Translational Medical Science at the University of Naples “Federico II”, and age- and sex-matched healthy controls with negative history for obesity and any form of malnutrition, visiting the Center because of minimal surgical procedures, were considered eligible for the study.

The exclusion criteria were: non-caucasian ethnicity; age < 6 or > 18 years; concomitant presence of chronic diseases, malignancies, immunodeficiencies, chronic infections, autoimmune diseases, chronic inflammatory bowel diseases, celiac disease, metabolic-genetic diseases, cystic fibrosis and other chronic lung diseases, cardiovascular/respiratory/gastrointestinal malformations, neuropsychiatric and neurological disorders; history of obesity surgery; presence of tattoos, scars, moles or lesions on both forearms.

### Outcomes

The study outcomes were the comparative evaluation of the dietary UPFs intake in pediatric patients with obesity and in sex- and age- matched healthy controls, and of the potential mechanisms elicited by UPFs in facilitating obesity focusing on the daily intake of the three major dietary AGEs [Nε -(carboxymethyl)lysine (CML), Nε -(1-carboxyethyl)lysine (CEL), and Nδ -(5-hydro-5- methyl-4-imidazolon-2-yl)-ornithine (MG-H1)], the AGEs skin accumulation, and their effects on mitochondrial metabolism. The participants daily intake of energy and nutrients were also assessed.

### Sample size calculation

Since “UFO” was a pilot study, sample size was not determined. An allocation ratio of 2:1 was used to adjust for case variability.

### Clinical and biochemical assessment

After collection of the written informed consent by the participants and their parents/legal guardian, we collected data regarding anamnestic and clinical features, personal and anthropometric data, gestational age, mode of delivery, birth weight, breastfeeding, breastfeeding duration, weaning age, maternal smoking during pregnancy and lactation, living setting, parents/legal guardian educational level of all participants. Anthropometric measurements were performed by trained dieticians following a 12-h fasting. Body weight and height were measured with the participants dressed in light indoor clothing and without shoes using a calibrated mechanical column scale. BMI were calculated as weight (in kilograms) divided by height (in meters) squared. BMI percentile for sex and age were calculated using the World Health Organization (WHO) growth charts^40^.

### Nutritional assessment

The 7-day (5 weekdays, 2 weekend days) weighed food diaries were used to investigate the UPFs intake and the daily intake of the three dietary AGEs (CML, CEL, MG-H1). Participants and their parents/legal guardian received complete oral and written instructions about how to weigh food and to record such data by experienced pediatric dietitians. They were requested to provide any appropriate additional information, for example, method of cooking and names of food brands. The food diaries section included the name of meals (breakfast, lunch, dinner, between-meal eating); mealtime; names of dishes (i.e., sushi); names of foods or ingredients in the dishes (i.e., rice, tuna, soya sauce); measured weight of each ingredient, food and/or meal; and place of meal consumption. Food diaries were revised by dietitians with parents/legal guardians and children to increase accuracy and completeness of reporting.

All foods and ingredients consumed by the participants were classified according to the NOVA classification^41^. The NOVA system considers groups of foods and drinks according their industrial processing and the use of additives. This system distinguishes four groups. All foods and ingredients consumed by the subjects were classified into one of the following four categories indicating levels of industrial food processing:

1. The first NOVA group (G1) is formed by “unprocessed or minimally processed foods”—fresh foods or foods that are minimally altered by industrial processes (drying, freezing, vacuum packaging, non-alcoholic fermentation), but free of added ingredients (i.e., fresh or frozen fruits and vegetables, eggs, pasteurized milk, meat, seeds, nuts, grains or plain yogurt);
2. The second group (G2) includes “processed culinary ingredients”—substances obtained directly from G1 foods or from nature, and used in preserving, cooking and seasoning (i.e., oils, fats, sugar and salt);
3. The third group (G3) is of “processed foods”—industrial products made by adding substance from G2 into foods from G1, using preservation methods such as canning and bottling, smoking, curing, or fermentation (i.e., canned vegetables, canned fish, fruits in syrup, cheeses, fresh bread, beer and wine);
4. The fourth group (G4) includes “ultra-processed food and drink products”, formulations manufactured using several ingredients, including little or no fresh foods, and a series of processes that greatly breakdown the food matrix. Processes and ingredients used in manufacturing are designed to create highly convenient, attractive and profitable products (i.e., soft drinks, sweet or savory packed snacks, processed meats, pre-prepared frozen dishes and ‘instant’ products).

The following foods were considered to be UPFs: instant and canned soups; reconstituted meat and fish products; ready-made sauces, gravies and dressings; French fries and other pre-made potato products such as chips; ready-to-eat and dry-mix desserts such as pudding; confectionery; sweet and savoury snack foods including granola bars and protein bars; sugar-sweetened or artificially sweetened beverages including soda, fruit drinks, pre-sweetened tea and coffee, energy drinks and dairy-based drinks; flavoured and/or sweetened yoghurt; industrially manufactured cakes, cookies and pies; dry cake and pancake mixes; industrially manufactured breads; sweet breakfast cereals; frozen and shelf-stable plate meals; ice cream, frozen yogurt and ice pops; meatless patties and fish sticks and infant formula.

Subjects’ UPFs consumption were calculated summing the amount consumed of food and beverage (g/day) included in the fourth category of the NOVA classification (G4), and then were calculated the proportion (%) of UPFs in the total weight of food and beverages consumed (in g/day) [daily intake of NOVA fourth group of food and beverages in grams/total daily food and beverages intake in grams *100], as previously reported^42^. In addition, the daily energy deriving from UPFs (in Kcal) was calculated using an ad hoc software (Winfood, Medimatica Srl, Italy).

Furthermore, specifically designed software (Winfood, Medimatica Srl, Italy) was also used to estimate the total daily intake of energy, macro- and micronutrients.

Finally, to estimate the daily dietary AGEs intake from the food diaries, a reference database of > 200 food products commonly consumed in a Western diet built at the Maastricht University (Maastricht, The Netherlands) was used^33^. The reference database can be found as Supplementary Table S1 online. This database reports the content of 3 major AGEs, CML, CEL and MG-H1, in mg per 100 g of food, quantified in the protein fractions of food products using highly specific ultra-performance liquid chromatography tandem mass spectrometry. All these data were recorded in a dedicated clinical chart.

### Assessment of skin AGEs accumulation

Selected AGEs have structural properties that cause them to emit fluorescent light across a specific range of wave-lengths upon excitation by ultraviolet light^43^. This unique characteristic has been used to develop a non-invasive device (AGEs Reader®; DiagnOptics Technologies, Groningen, The Netherlands), that quantifies accumulated AGEs within the human skin through the evaluation of sAF as previously described^44,45^. For the measurement, the participants were asked to place the dominant forearm on the device for 60 s. The AGE Reader® illuminated a skin area of 4 cm^2^ with ultraviolet A light with a single peak excitation wavelength of 370 nm. Emission light (fluorescence in the wavelength of 420–600 nm) and reflected excitation light (with a wavelength of 300–420 nm) from the skin were measured using a spectrometer. Skin AGEs levels were calculated as the ratio between the emission light and reflected excitation light, multiplied by 100 and expressed in arbitrary units (AU). The intra- and inter-day coefficient was 2.6%. In all children, skin oils/ointments were not recently applied on the site of measurement before sAF measurement. Three measurements were performed on different sites of the skin on the volar side of the dominant forearm, as previously described^46^. The mean sAF value of the three measurements was reported in the participants’ clinical chart.

### Isolation of peripheral mononuclear blood cells

Peripheral mononuclear blood cells (PBMCs) were collected from a randomly selected subgroup of obese pediatric patients and well sex- and age- matched healthy controls. PBMCs were obtained from 10 obese patients (50% male, mean age 11 years ± 2.8 SD) and 10 age- and sex-matched healthy controls (50% male, mean age 11 years ± 2.8 SD) and were isolated by Ficoll density gradient centrifugation (Ficoll-Histopaque − 1077, Sigma, St. Louis, Missouri, USA). Briefly, cells were stratified on 3 mL of Ficoll and centrifuged 15 min at 1000×g at room temperature. After centrifugation, the opaque interface containing mononuclear cells was carefully aspirated with a Pasteur pipette and cells were washed with 10 mL of PBS and centrifuged 10 min at 500×g at room temperature. After centrifugation, the upper layer was discarded and PBMCs (3.5 × 10^5^ cells/well) were cultured in duplicates in 96-well plates in 200 µl culture medium (RPMI 1640, Gibco, ThermoFisher, Waltham, Massachusetts, USA) containing 10% FBS (Gibco), 1% non-essential amino acids (Gibco), 1% sodium pyruvate (Gibco), and 1% penicillin/streptomycin (Gibco).

### PBMCs stimulation protocol

PBMCs (3.5 × 10^5^cells/well) from healthy children were stimulated with 200 µg/mL of AGE-BSA (#ab51995, Abcam, Cambridge, UK; purity: > 98%; endotoxin level: < 0.100 Eu/µg) or BSA (Sigma-Aldrich, Missouri, USA), as control for 48 h. Timing and dosing were selected based on the results of previous time-course and dose response experiments performed at our laboratory. The cells with only medium were used as negative control. All the experiments were performed in triplicate.

### Seahorse XFp analyses to assess mitochondrial metabolism

Mitochondrial metabolism in PBMCs were assessed by the Seahorse XFp analyzer (Seahorse Biosciences, North Billerica, MA, USA), by using the Cell Mito Stress Test kit (cat# 103,010–100). Before cell mito stress analyses, the cells were centrifuged at room temperature at 1200 rpm for 10 min, the medium was replaced with a buffered base medium (Agilent Seahorse-103193) supplemented with 2 mM glutamine, 1 mM pyruvate and 10 mM glucose at pH 7.4. In order to compare the mitochondrial metabolism of PBMCs from healthy controls and obese patients, PBMCs were seeded (3.5 × 105cells/well) in Seahorse mini-plates in enriched buffered base medium (Agilent Seahorse-103193). The plates were centrifuged at 200 g for 5 min at room temperature and equilibrated at 37 °C in a CO2 free incubator for at least 1 h. Basal oxygen consumption rate (OCR) was determined in the presence of glutamine (2 mM) and pyruvate (1 mM). The proton leak was determined after inhibition of mitochondrial ATP production by 1 µM oligomycin, as an inhibitor of the F0–F1 ATPase. The measurement of the ATP production in the basal state was obtained from the decrease in respiration by inhibition of the ATP synthase with oligomycin. The mitochondrial electron transport chain was stimulated maximally by the addition of the uncoupler FCCP (1 µM). Coupling efficiency is the proportion of the oxygen consumed to drive ATP synthesis compared with that driving proton leakage and was calculated as the fraction of basal mitochondrial OCR used for ATP synthesis (ATP-linked OCR/basal OCR). Spare respiratory capacity (SRC) is the capacity of the cell to respond to an energetic demand and was calculated as the difference between the maximal respiration and basal respiration. The mitochondrial respiration was expressed as the oxygen consumption rate per min normalized to the number of cells. The same cell number/well was plated before the OCR measurements; the cell count was obtained by using the Burker chamber. In addition, at the end of analyses, we determined the protein content/well by Bradford assay without observing significant differences.

### Oxidative stress biomarkers assessment

Plasmatic concentrations of GSH and GSSG were measured with the dithionitrobenzoic acid-GSSG reductase recycling assay. The GSH/GSSG ratio has been performed.

### Data quality assurance

All data were recorded anonymously. At the study Center, designated investigators were required to enter all collected data in the case report form (CRF). Two researchers performed separate checks of data completeness, clarity, consistency, and accuracy, and instructed site personnel to make any required corrections or additions. Using a single data-entry method, all data recorded in the CRF were entered in the study database by the same researcher. Then, the study dataset was reviewed and underwent data cleaning and verification according to standard procedures. Finally, the database was locked once it was declared complete and accurate, and the statistical analysis was performed.

### Statistical analysis

Results were expressed as mean ± SD for continuous variables and as median and 95% cluster confidence intervals for variables with a non-Gaussian distribution. Discrete variables were reported as numbers and proportions. Differences between obese patients *vs.* healthy controls were tested by unpaired t-test. Data from mitochondrial metabolism experiments were evaluated by ANOVA followed by the Newman-Keuls post hoc test to correct for multiple comparisons. Differences were considered statistically significant at *p* < 0.05. All analyses were performed using GraphPad Prism 7.0 (GraphPad Software, San Diego, CA, USA).

### Supplementary Information

Supplementary Table S1.

## Data Availability

The dataset used and/or analyzed during the current study is available from the corresponding author on reasonable request.
