# Supplementary material for: Increased dietary intake of ultraprocessed foods and mitochondrial metabolism alterations in pediatric obesity
Source: Sci Rep. 2023 Aug 3;13:12609. doi: 10.1038/s41598-023-39566-9 (PMC10400566; doi:10.1038/s41598-023-39566-9)
Supplement: Supplementary file 1 — Supplementary Table S1. [file 41598_2023_39566_MOESM1_ESM.docx]

**Increased Dietary Intake of Ultraprocessed Foods and Mitochondrial Metabolism Alterations in Pediatric Obesity**

**Serena Coppola^1,2^, Lorella Paparo^1,2^, Giovanna Trinchese^3^, Andrea Margarita Rivieri^1^, Antonio Masino^1,2^, Anna Fiorenza De Giovanni Di Santa Severina^1,2^, Mariapina Cerulo^1^, Maria Escolino^1^, Assunta Turco^1^, Ciro Esposito^1^, Maria Pina Mollica^3^, and Roberto Berni Canani^1,2,3,4*^**

*^1^Department of Translational Medical Science, University Federico II, Naples, Italy*

*^2^ImmunoNutritionLab at CEINGE Advanced Biotechnologies, University Federico II, Naples, Italy*

*^3^European Laboratory for the Investigation of Food-Induced Diseases, University Federico II, Naples, Italy*

*^4^Task Force for Microbiome Studies, University Federico II, Naples, Italy*

*^5^Department of Biology, University Federico II, Naples, Italy*

# *** Corresponding Author:

Prof. Dr. Roberto Berni Canani, MD, PhD

P.: +39 0817462680

[Email: berni@unina.it](mailto:Email:%20berni@unina.it)

**Supplementary Table S1.** The reference database used to estimate the daily dietary AGEs intake.

| # | Food | Specification | Source | mg CML/100g | mg CEL/100g | mg MGH1/100g |
| --- | --- | --- | --- | --- | --- | --- |
|  | *Potatoes* | | | | | |
| 1 | Chips | Deep fried 5 minutes | Supermarket | 0.14 | 0.09 | 1.28 |
| 2 | Potato-boiled | Boiled, 7 minutes | Supermarket | 0.01 | 0.00 | 0.39 |
| 3 | Potato-fried | Boiled, 7 minutes, fried 6 minutes | Supermarket | 0.01 | 0.01 | 0.30 |
|  | *Alcoholic and non-alcoholic beverages* | | | | | |
| 4 | Coffee |  | Canteen university | 0.00 | 0.01 | 0.24 |
| 5 | Red wine |  | Restaurant, regular bottle | 0.00 | 0.00 | 0.00 |
| 6 | Fruit juice | Kiwi/Orange | Supermarket, Hero | 0.00 | 0.00 | 0.05 |
| 7 | Cola |  | Supermarket, Coca Cola | 0.00 | 0.00 | 0.00 |
|  | *Bread* | | | | | |
| 8 | Wholemeal bread |  | Supermarket | 0.28 | 0.21 | 3.36 |
| 9 | Brown bread |  | Supermarket | 0.40 | 0.31 | 6.01 |
| 10 | Rye bread | Dark, brand A | Supermarket | 0.90 | 0.09 | 2.57 |
| 12 | Rye bread | Dark, brand B | Supermarket | 0.58 | 0.18 | 2.74 |
| 14 | Rye bread | Light | Supermarket | 0.59 | 0.18 | 5.22 |
| 15 | White bread (n=3)^*^ |  | Supermarket | 0.24 ± 0.02 | 0.11 ± 0.02 | 2.55 ± 0.33 |
| 16 | White bread (n=3)^*^ | Belgian made | Supermarket | 0.21 ± 0.05 | 0.11 ± 0.01 | 2.48 ± 0.19 |
| 17 | White bread (n=3)^*^ | German made | Supermarket | 0.31 ± 0.01 | 0.13 ± 0.01 | 2.09 ± 0.15 |
| 18 | White bread (n=3)^*^ | Dutch made | Supermarket | 0.13 ± 0.02 | 0.07 ± 0.01 | 2.18 ± 0.04 |
| 19 | Currant bread |  | Supermarket | 1.11 | 0.56 | 3.35 |
| 20 | Luxurious breads/buns | Baguette, oven heated 9 minutes, 200 C | Supermarket, vacuum | 0.22 | 0.10 | 2.28 |
| 21 | Croissant |  | Supermarket, fresh | 1.04 | 1.33 | 9.89 |
| 22 | Rusk |  | Supermarket | 1.96 | 1.44 | 23.1 |
| 23 | Knäckebröd |  | Supermarket | 0.96 | 1.41 | 13.5 |
| 24 | Toast (bread) | 2½ minutes in toaster | Supermarket | 0.52 | 0.55 | 4.16 |
| 25 | Toast (cracker) |  | Supermarket | 2.31 | 0.76 | 9.97 |
| 26 | Baguette, toasted | 2½ minutes in toaster | Supermarket, fresh | 0.23 | 0.10 | 3.61 |
| 27 | White bread without crust | Belgian made | Supermarket | 0.21 | 0.03 | 1.86 |
| 28 | Wholemeal bread without crust |  | Supermarket | 0.24 | 0.05 | 4.10 |
| 29 | Pita bread | Oven heated, 15 min, 175 C | Supermarket, vacuum | 0.25 | 0.06 | 2.82 |
|  | *Eggs* | | | | | |
| 30 | Egg yolk | Boiled, 10 minutes | Supermarket | 0.06 | 0.08 | 0.08 |
| 31 | Egg white | Boiled, 10 minutes | Supermarket | 0.10 | 0.07 | 0.35 |
| 32 | Fried egg | Fried, 4 minutes | Supermarket | 0.42 | 0.52 | 4.50 |
|  | *Fruits* | | | | | |
| 33 | Kaki |  | Supermarket | 0.00 | 0.00 | 0.00 |
| 34 | Banana |  | Supermarket | 0.01 | 0.00 | 0.05 |
| 35 | Apple sauce | Light | Supermarket, Hak | 0.00 | 0.00 | 0.20 |
| 36 | Olives | Green | Restaurant | 0.12 | 0.01 | 1.54 |
|  | *Pastry and biscuits* | | | | | |
| 37 | Dutch spiced cake (n=3)^*^ | Dutch made (Batch 1) | Supermarket | 0.79 ± 0.05 | 1.22 ± 0.09 | 20.31 ± 0.82 |
| 38 | Dutch spiced cake (n=3)^*^ | Dutch made (Batch 2) | Supermarket | 0.91 ± 0.02 | 1.13 ± 0.27 | 19.76 ± 1.81 |
| 39 | Dutch spiced cake (n=3)^*^ | Belgian made (Batch 1) | Supermarket | 0.65 ± 0.09 | 0.84 ± 0.19 | 12.97 ± 2.42 |
| 40 | Dutch spiced cake (n=3)^*^ | Belgian made (Batch 2) | Supermarket | 0.74 ± 0.17 | 0.84 ± 0.27 | 15.87 ± 2.12 |
| 42 | Dutch spiced cake (n=3)^*^ | German made (Batch 1) | Supermarket | 0.79 ± 0.11 | 0.91 ± 0.08 | 8.07 ± 0.14 |
| 44 | Dutch spiced cake (n=3)^*^ | German made (Batch 2) | Supermarket | 0.91 ± 0.08 | 1.24 ± 0.33 | 10.87 ± 0.78 |
| 46 | Apfelstrudel | Oven heated 40 minutes, 200 C | Supermarket, frozen | 0.07 | 0.05 | 0.97 |
| 47 | Apricot pie |  | Supermarket | 0.25 | 0.47 | 3.47 |
| 48 | Cream pie |  | Supermarket | 0.48 | 0.03 | 1.14 |
| 49 | Tompouce |  | Supermarket, fresh | 0.27 | 0.24 | 2.23 |
| 50 | Pancake | With mascarpone | Restaurant | 0.49 | 0.17 | 1.38 |
| 51 | Cake |  | Supermarket | 1.75 | 0.23 | 2.61 |
| 52 | Syrup waffle | *"Stroopwafel"* | Supermarket | 0.46 | 0.58 | 11.31 |
| 53 | Cake | With almond paste | Supermarket | 0.51 | 0.55 | 5.57 |
| 54 | Biscuit | Multigrain | Supermarket | 1.33 | 1.06 | 19.62 |
| 55 | Biscuit | With peanuts | Supermarket | 2.59 | 3.42 | 36.90 |
| 56 | Biscuit | Speculoos | Supermarket | 2.47 | 1.99 | 28.31 |
| 57 | Biscuit | With currants and cherry | Supermarket, Sultana | 1.03 | 0.48 | 3.99 |
| 58 | Rice cracker | With cheese powder | Supermarket | 1.13 | 1.04 | 11.34 |
| 59 | Rice cracker | With sea salt | Supermarket | 0.91 | 0.75 | 8.01 |
|  | *Cereals and cereal products* | | | | | |
| 60 | Cereals | Honey loops | Supermarket, Kellogg | 1.20 | 0.70 | 3.60 |
| 61 | Cereals | Honey granola | Supermarket, Dorsett | 1.04 | 1.02 | 9.44 |
| 62 | Cereals | Crunchy muesli | Supermarket, Quaker | 0.46 | 0.46 | 8.61 |
| 63 | Cereals (n=8)^*^ | Cornflakes | Supermarket, Kellogg | 0.66 ± 0.08 | 0.79 ± 0.13 | 12.12 ± 3.53 |
| 64 | Cereals | Puffed wheat | Supermarket, Euroshopper | 1.27 | 0.84 | 15.61 |
| 65 | Cereals | Frosted flakes | Supermarket, Kellogg | 0.63 | 0.53 | 25.89 |
| 66 | Cereals | Rice crispies | Supermarket, Kellogg | 0.59 | 0.47 | 14.70 |
| 67 | Cereals | Cooked oats | Supermarket, Quaker | 1.17 | 0.43 | 11.31 |
| 68 | Cereals | Miniwheats | Supermarket, Weetabix | 1.96 | 1.64 | 41.60 |
| 69 | Rice, boiled | Basmati | Supermarket, dried | 0.07 | 0.02 | 1.37 |
| 70 | Rice, fried | Boiled, then fried | Supermarket, dried | 0.96 | 1.32 | 8.59 |
| 71 | Rice, fried | Fried in olive oil | Supermarket, dried | 0.12 | 0.03 | 4.17 |
| 72 | Pasta, boiled | Penne | Supermarket, dried | 0.59 | 0.11 | 2.51 |
| 73 | Pasta, boiled | Spaghetti | Supermarket, dried | 0.82 | 0.13 | 3.02 |
| 74 | Pasta, boiled | Macaroni | Supermarket, dried | 0.43 | 0.06 | 1.41 |
|  | *Vegetables* | | | | | |
| 75 | Mushroom | Fried, 5 minutes | Supermarket | 0.01 | 0.01 | 0.01 |
| 76 | Peas | Boiled | Supermarket, frozen | 0.05 | 0.06 | 2.31 |
| 77 | Tomato | Fried, 3 minutes | Supermarket | 0.07 | 0.05 | 0.65 |
| 78 | Tomato, canned |  | Supermarket, canned | 0.03 | 0.01 | 0.11 |
|  | *Savoury bread spreads* | | | | | |
| 79 | Peanut butter (n=3)^*^ | Pâte de cacahouètes, 72% peanut | Supermarket | 1.91 ± 0.23 | 5.56 ± 0.31 | 35.62 ± 2.51 |
| 80 | Peanut butter (n=3)^*^ | Delvita Erdnuss Crème, 90% peanut | Supermarket | 3.11 ± 0.31 | 6.74 ± 0.45 | 44.52 ± 2.03 |
| 81 | Peanut butter (n=3)^*^ | Calvé, 85% peanut | Supermarket | 2.63 ± 0.68 | 6.85 ± 0.68 | 42.05 ± 4.08 |
| 82 | Peanut butter (n=8)^*^ | Plus, 90% peanut | Supermarket | 1.38 ± 0.07 | 3.74 ± 0.25 | 20.39 ± 5.09 |
|  | *Cheese* | | | | | |
| 83 | Cheese | Gouda 48+ (32% fat) | Supermarket | 0.03 | 0.01 | 0.08 |
| 84 | Cheese | (32% fat) | Supermarket | 0.04 | 0.08 | 0.25 |
| 85 | Cheese | Old (35% fat) | Supermarket | 0.08 | 0.22 | 0.21 |
| 86 | Parmesan cheese | Parmigiano Reggiano (28% fat) | Supermarket | 0.22 | 0.63 | 2.76 |
| 87 | Cheese | (32% fat) | Supermarket | 0.15 | 0.55 | 0.30 |
| 88 | Cream cheese | (7% fat) | Supermarket, Slankie | 0.35 | 0.08 | 0.49 |
| 89 | French cheese | Brie, Coeur de Lion, 60+ (32% fat) | Supermarket | 0.01 | 0.00 | 0.07 |
| 90 | French cheese | Camembert, Chêne d'argent (21% fat) | Supermarket | 0.12 | 0.30 | 0.16 |
| 91 | Cottage cheese |  | Supermarket | 0.05 | 0.04 | 0.12 |
| 92 | Mozzarella |  | Supermarket | 0.07 | 0.01 | 0.13 |
| 93 | Feta cheese |  | Supermarket | 0.08 | 0.47 | 1.14 |
|  | *Milk and milk products* | | | | | |
| 94 | Milk | Semi-skimmed | Supermarket | 0.25 | 0.02 | 0.12 |
| 95 | Milk | Skimmed | Supermarket, fresh | 0.07 | 0.01 | 0.08 |
| 96 | Milk | Skimmed | Supermarket | 0.26 | 0.02 | 0.30 |
| 97 | Milk | Skimmed, microwave 2 min | Supermarket, Optimel | 0.36 | 0.02 | 0.38 |
| 98 | Yoghurt drink | 0% fat, raspberry | Supermarket, Optimel | 0.04 | 0.03 | 0.12 |
| 99 | *"Kwark"* | Curd, skimmed | Supermarket | 0.06 | 0.04 | 0.26 |
| 100 | Milk | Whole | Supermarket, fresh | 0.01 | 0.01 | 0.02 |
| 101 | Evaporated milk |  | Supermarket | 0.66 | 0.03 | 0.09 |
| 102 | Evaporated milk | Skimmed | Supermarket | 0.47 | 0.08 | 0.38 |
| 103 | Evaporated milk | Semi-skimmed | Supermarket | 2.23 | 0.12 | 0.54 |
| 104 | Whipped cream | 35% fat | Supermarket, liquid | 0.00 | 0.00 | 0.00 |
| 105 | Chocolate milk | Whole | Supermarket | 0.38 | 0.20 | 0.66 |
| 106 | Chocolate milk | Semi-skimmed | Supermarket | 1.01 | 0.26 | 0.98 |
| 107 | Custard | *"Vla"*, chocolate/vanilla | Supermarket | 0.12 | 0.06 | 0.43 |
| 108 | Porridge | Rolled oats | Supermarket, MelkUnie | 0.02 | 0.00 | 0.06 |
| 109 | Porridge | Rice | Supermarket, MelkUnie | 0.03 | 0.02 | 0.21 |
| 110 | Pudding | Raspberry, with strawberry sauce | Supermarket, Mona | 0.26 | 0.02 | 0.33 |
| 111 | Yogurt | Semi-skimmed, vanilla | Supermarket | 0.01 | 0.01 | 0.13 |
| 112 | Ice-cream | Vanilla/Strawberry | Supermarket, frozen | 0.00 | 0.00 | 0.03 |
|  | *Nuts, seeds and snacks* | | | | | |
| 113 | Pistachios | Unprocessed | Supermarket | 0.21 | 0.09 | 0.32 |
| 114 | Pecans | Roasted, salted | Supermarket | 1.06 | 1.95 | 16.63 |
| 115 | Peanuts | In shell | Store | 1.37 | 2.43 | 25.71 |
| 116 | Peanuts | Salted | Supermarket | 1.72 | 3.39 | 26.59 |
| 117 | Cocktail nuts | *"Borrelnoten"* Italian herbs | Supermarket, Duyvis | 1.05 | 0.82 | 6.82 |
| 118 | Cocktail nuts | *"Borrelnoten"* Sateh curry | Supermarket, Duyvis | 1.58 | 2.22 | 12.34 |
| 119 | Cocktail nuts | *"Borrelnoten"* Spicy | Supermarket, Perfekt | 0.55 | 1.13 | 8.06 |
| 120 | Potato crisps | Kettle | Supermarket | 0.23 | 0.27 | 1.63 |
| 121 | Corn crisps | Vitasia Tikka Masala | Supermarket | 0.29 | 0.11 | 2.79 |
| 122 | *"Kroket"* | Beef filling, deep fried 7,5 minutes | Supermarket, frozen | 0.41 | 0.44 | 4.06 |
| 123 | Sausage roll | Oven heated, 8 minutes | Supermarket, fresh | 1.01 | 0.45 | 5.26 |
| 124 | Satay | Pork, with peanut sauce, microwave heated 60 seconds | Supermarket, vacuum | 0.45 | 0.90 | 5.70 |
| 125 | *"Frikandel"* | Deep fried 7,5 minutes | Supermarket, frozen | 0.99 | 1.42 | 5.07 |
|  | *Mixed dishes* | | | | | |
| 126 | Spring roll | Deep fried, with vegetables and meat | Restaurant | 0.20 | 0.07 | 1.53 |
| 127 | Pizza | Mozzarella pesto, oven heated 12 minutes 175 C | Supermarket, frozen | 0.06 | 0.05 | 0.95 |
| 128 | Russian salad | With beef, potato, onion, peas, mayonnaise | Supermarket | 0.13 | 0.03 | 0.67 |
|  | *Soups* | | | | | |
| 129 | Tomato soup, canned | Microwave heated, 1,5 minutes 800 Watt | Supermarket, vacuum | 0.00 | 0.00 | 0.00 |
| 130 | Pea soup | Home made, heated | Self-made | 0.04 | 0.11 | 2.23 |
|  |  |  |  |  |  |  |
|  | *Soy products and vegetarian products* | | | | | |
| 131 | Vegetarian burger | Vegetable burger, fried 5 minutes | Supermarket | 0.42 | 0.60 | 3.60 |
| 132 | Tofu | Fried 10 minutes | Supermarket | 0.94 | 0.74 | 11.87 |
| 133 | Tempe | Boiled, 10 minutes | Supermarket | 0.47 | 0.29 | 3.50 |
| 134 | Tempe | Grilled, 4 minutes | Supermarket | 1.00 | 0.59 | 6.87 |
| 135 | Soy sauce | Ketjap manis | Supermarket | 0.01 | 0.02 | 0.05 |
|  | *Sugar, sweets and sweet sauces* | | | | | |
| 136 | Praline | With orange crème filling | Supermarket | 0.41 | 0.09 | 0.87 |
| 137 | Chocolate | White | Supermarket | 0.52 | 0.06 | 0.42 |
| 138 | Chocolate | Milk | Supermarket | 0.96 | 0.31 | 1.33 |
| 139 | Chocolate | Milk, with hazelnuts | Supermarket | 1.33 | 0.55 | 3.23 |
| 140 | Chocolate | Dark | Supermarket | 3.51 | 1.28 | 3.03 |
| 141 | Mars (n=8)^*^ |  | Supermarket | 0.70 ± 0.03 | 0.36 ± 0.02 | 0.15 ± 0.03 |
| 142 | Twix |  | Supermarket | 0.93 | 0.81 | 5.83 |
| 143 | Bounty |  | Supermarket | 0.71 | 0.23 | 2.16 |
| 144 | Milky Way |  | Supermarket | 1.68 | 0.56 | 0.54 |
| 145 | Liquorice |  | Supermarket | 0.06 | 0.02 | 0.08 |
| 146 | Candy | Jelly bears | Supermarket | 0.03 | 0.02 | 0.11 |
| 147 | Jam | Blackberry | Supermarket | 0.00 | 0.00 | 0.00 |
| 148 | *"Hagelslag"* | Chocolate sprinkles | Supermarket | 5.09 | 2.03 | 9.33 |
| 149 | *"Hagelslag"* | Fruit sprinkles | Supermarket | 0.00 | 0.00 | 0.00 |
| 150 | *"Stroop"* | Apple syrup | Supermarket | 0.05 | 0.02 | 0.44 |
| 151 | Chocolate spread |  | Supermarket | 0.90 | 0.34 | 3.27 |
|  | *Fats, oils and savoury sauces* | | | | | |
| 152 | Peanut sauce | Microwave heated, 0.5 minutes, 800 Watt | Supermarket, vacuum | 0.21 | 0.33 | 1.90 |
| 153 | Peanut sauce | Microwave heated, 1.5 minutes, 800 Watt | Supermarket, vacuum | 0.65 | 0.95 | 6.94 |
| 154 | Peanut sauce | Instant, 90% peanut, 1:1 made up with boiling water | Supermarket, instant | 1.29 | 2.35 | 14.03 |
| 155 | Peanut sauce | Pan heated, 500g with 300ml water | Supermarket, vacuum | 0.80 | 1.39 | 12.60 |
| 156 | Tomato sauce | Pan heated | Supermarket | 0.04 | 0.05 | 0.22 |
| 157 | Curry sauce | Instant, 1:10 made up with boiling water | Supermarket, instant | 0.05 | 0.01 | 0.13 |
| 158 | Cheese-sauce | Instant, 1:6 made up with boiling water | Supermarket, instant | 0.06 | 0.02 | 0.18 |
| 159 | Mayonnaise |  | Supermarket | 0.01 | 0.00 | 0.12 |
| 160 | Frying butter | Liquid, pan heated | Supermarket | 0.00 | 0.00 | 0.00 |
| 161 | Butter | Diet (60% fat) | Supermarket | 0.00 | 0.00 | 0.00 |
| 162 | Olive oil | Pan heated | Supermarket | 0.00 | 0.00 | 0.00 |
| 163 | Ketchup |  | Supermarket | 0.05 | 0.01 | 0.05 |
| 164 | Garlic sauce |  | Supermarket | 0.01 | 0.00 | 0.02 |
|  | *Fish* | | | | | |
| 165 | Salmon, canned | In brine | Supermarket, canned | 1.17 | 2.82 | 10.93 |
| 166 | Salmon, fried | Fried, 10 minutes | Supermarket | 0.41 | 0.58 | 1.83 |
| 167 | Salmon, smoked |  | Supermarket, vacuum | 0.58 | 0.22 | 0.32 |
| 168 | Herring | Raw | Supermarket, vacuum | 0.06 | 0.06 | 0.09 |
| 169 | Hake | Breaded, oven heated, 10 minutes 200 C | Supermarket | 0.38 | 0.31 | 0.55 |
| 170 | Whitefish | Grilled, in soy sauce | Restaurant | 0.26 | 0.29 | 1.08 |
| 171 | Prawns | Fried, 5 minutes | Restaurant | 0.06 | 0.07 | 1.03 |
| 172 | Tuna, canned | In olive oil | Supermarket, canned | 0.35 | 1.26 | 3.78 |
| 173 | Cod | Fillet, fried 8 minutes | Supermarket | 0.13 | 0.11 | 0.66 |
| 174 | Steamed fish | Oven heated, 15 minutes 175 C | Supermarket | 0.05 | 0.06 | 0.11 |
| 175 | Fish fingers | Oven heated, 15 minutes 175 C | Supermarket, frozen | 0.30 | 0.18 | 4.30 |
|  | *Meats, meat products and poultry* | | | | | |
| 176 | Minced beef (10% fat) | Beef, fried, 8 minutes | Supermarket | 0.37 | 0.44 | 0.54 |
| 177 | Red cooked beef (n=3)^*^ | Stewed, 2,5 hours in wine vinegar, with garlic and star aniseed | Supermarket | 2.03 ± 0.45 | 5.63 ± 1.12 | 13.48 ± 6.01 |
| 178 | Minced beef (n=2)^*^ | Steamed, 15 min | Supermarket | 0.44 ± 0.11 | 0.25 ± 0.11 | 2.06 ± 1.38 |
| 179 | Minced beef (n=2)^*^ | Fried, 10 minutes | Supermarket | 0.51 ± 0.08 | 0.31 ± 0.10 | 0.86 ± 0.24 |
| 180 | Beef steak | Grilled 5 minutes | Supermarket | 0.73 | 0.40 | 0.90 |
| 181 | Roast beef | Oven heated, 1 hour 150 C | Supermarket | 1.01 | 0.41 | 2.09 |
| 182 | Beef steak (n=3)^*^ | Fried, 4 minutes | Supermarket | 0.12 ± 0.08 | 0.23 ± 0.11 | 0.34 ± 0.12 |
| 183 | Beef steak (canned) (n=3)^*^ | Pan heated, 10 minutes | Supermarket, canned | 1.07 ± 0.38 | 5.63 ± 2.01 | 11.96 ± 2.78 |
| 184 | Pork, shoulder chops (n=3)^*^ | With garlic, fried 12 minutes | Supermarket | 0.15 ± 0.04 | 0.35 ± 0.17 | 0.51 ± 0.24 |
| 185 | Pork (n=3)^*^ | Fried, 3 minutes, then stewed, 40 minutes | Supermarket | 0.61 ± 0.12 | 1.03 ± 0.28 | 1.71 ± 0.53 |
| 186 | Pork fillet roulade | Oven heated, 40 min 175 C | Supermarket | 0.11 | 0.15 | 0.49 |
| 187 | Minced beef (21% fat) | 50/50 beef and pork, fried 5 minutes | Supermarket | 0.94 | 1.68 | 2.19 |
| 188 | *"Slavink"* | Minced beef wrapped in bacon, fried, 20 minutes | Supermarket | 0.14 | 0.09 | 0.77 |
| 189 | Sausage | Fried, 13 minutes | Supermarket | 0.84 | 1.63 | 3.85 |
| 190 | Fried bacon | Fried | Restaurant | 0.72 | 1.82 | 3.01 |
| 191 | Pork | Strips, fried 7 minutes | Supermarket | 1.12 | 1.15 | 1.90 |
| 192 | Frankfurter, canned (n=2)^*^ |  | Supermarket, canned | 4.22 ± 0.16 | 1.01 ± 0.09 | 4.74 ± 0.68 |
| 193 | Frankfurter, canned (n=2)^*^ | Oven heated, 15 min 175 C | Supermarket | 3.33 ± 0.98 | 1.09 ± 0.22 | 4.22 ± 0.67 |
| 194 | Meat ball |  | Supermarket, canned | 0.83 | 0.90 | 4.61 |
| 195 | Meat ball | Oven heated, 15 min 175 C | Supermarket | 1.27 | 1.91 | 6.95 |
| 196 | Roasted pork | Roasted | Restaurant | 0.41 | 0.29 | 3.55 |
| 197 | Pork tenderloin (n=3)^*^ | Fried, 16 minutes | Supermarket | 0.34 ± 0.25 | 1.39 ± 1.14 | 3.23 ± 2.27 |
| 198 | Gammon steak (n=3)^*^ | Fried, 25 minutes | Supermarket | 0.22 ± 0.07 | 0.89 ± 0.61 | 1.04 ± 0.34 |
| 199 | Schnitzel (n=3)^*^ | Non-breaded, fried 6 minutes | Supermarket | 0.12 ± 0.05 | 0.44 ± 0.35 | 0.23 ± 0.16 |
| 200 | Rib eye | Fried, 15 minutes | Supermarket | 0.27 | 0.35 | 0.79 |
| 201 | Hamburger | Fried, 8 minutes | Supermarket | 0.20 | 0.29 | 1.58 |
| 202 | Black pudding | With crushed bacon, fried 16 minutes | Supermarket | 4.82 | 7.71 | 63.01 |
| 203 | Lamb, chops (n=3)^*^ | Fried, 6 minutes | Supermarket | 0.11 ± 0.03 | 0.30 ± 0.08 | 0.66 ± 0.22 |
| 204 | *"Rookworst"* (n=2)^*^ | Smoked sausage, beef, heated in hot water | Supermarket, vacuum | 0.98 ± 0.08 | 0.41 ± 0.18 | 1.06 ± 0.12 |
| 205 | *"Rookworst"* (n=2)^*^ | Smoked sausage, pork, heated in hot water | Supermarket, vacuum | 1.67 ± 1.27 | 0.46 ± 0.15 | 2.19 ± 0.27 |
| 206 | Gammon (boiled) | Cold cut | Supermarket | 0.12 | 0.33 | 0.37 |
| 207 | Ham, shoulder (boiled) | Cold cut | Supermarket | 0.32 | 0.66 | 1.26 |
| 208 | Steak tartare | Sweet onion | Supermarket | 0.08 | 0.04 | 0.21 |
| 209 | Roast beef | Cold cut | Supermarket | 0.28 | 0.41 | 0.44 |
| 210 | Salami | Cold cut | Supermarket | 1.27 | 0.58 | 3.15 |
| 211 | Salami sausage saveloy | Cold cut | Supermarket | 0.54 | 0.19 | 2.43 |
| 212 | Bacon rashers streaky | Cold cut | Supermarket | 2.54 | 0.93 | 0.71 |
| 213 | Drumsticks | With skin, oven heated, 40 minutes 200 C | Supermarket | 0.62 | 0.95 | 2.21 |
| 214 | Chicken Wings | With skin, oven heated | Supermarket | 0.82 | 0.78 | 3.04 |
| 215 | Ragout (chicken stew) | Pan heated | Supermarket, canned | 0.65 | 0.79 | 2.93 |
| 216 | Crusty chicken | Breaded | Restaurant | 0.75 | 0.73 | 4.05 |
| 217 | Chicken fillet | Fried, 30 minutes | Supermarket | 0.13 | 0.27 | 1.41 |
| 218 | Chicken (n=2)^*^ | Boiled, 30 minutes | Supermarket | 0.18 ± 0.03 | 0.14 ± 0.07 | 0.65 ± 0.07 |
| 219 | Chicken (n=2)^*^ | Fried, 10 minutes | Supermarket | 0.34 ± 0.02 | 0.26 ± 0.04 | 1.16 ± 0.41 |
| 220 | Chicken | Microwaved, 10 minutes | Supermarket | 0.92 | 1.02 | 5.16 |
| 221 | Pâté |  | Supermarket | 0.09 | 0.07 | 0.29 |
| 222 | Liverwurst (n=2)^*^ |  | Supermarket | 0.25 ± 0.14 | 0.30 ± 0.04 | 1.59 ± 0.41 |

*Data expressed as mean±sd
